# Glycometabolic Regulation of Angiogenesis: Mechanisms and Therapeutic Strategies

**DOI:** 10.3390/ijms26062386

**Published:** 2025-03-07

**Authors:** Zhifeng Yao, Junting Li, Jiaming Yu, Ye Cheng, Chang Fang, Xinlei Chen, Xiaoqi Chen, Yizheng Wang, Dong Gao, Fan Lin

**Affiliations:** 1 College of Integrative Medicine, Fujian University of Traditional Chinese Medicine, Fuzhou 350122, China; y1556269733@163.com (Z.Y.); 19859388022@163.com (J.L.); 18905907332@163.com (J.Y.);; 2 Key Laboratory of Integrative Medicine on Chronic Diseases, Fujian Province University, Fuzhou 350122, China

**Keywords:** angiogenesis, glycometabolism, glycolysis, clinical applications

## Abstract

Angiogenesis, the process by which new blood vessels emerge from pre-existing vasculature, forms the fundamental biological basis for therapeutic angiogenesis. In recent years, this field has garnered significant attention, particularly in the context of understanding the mechanisms of angiogenesis through the lens of glycometabolism. The potential clinical applications of this research have been widely acknowledged within the medical community. In this article, the role of angiogenesis and the principal molecular mechanisms that govern it are first delineated. The influence of glycometabolism on angiogenesis is then explored, with a focus on glycolysis. Finally, research on therapeutic angiogenesis based on the regulation of glycometabolism is presented, offering novel perspectives for ongoing research and clinical applications.

## 1. Introduction

**Figure 1:**
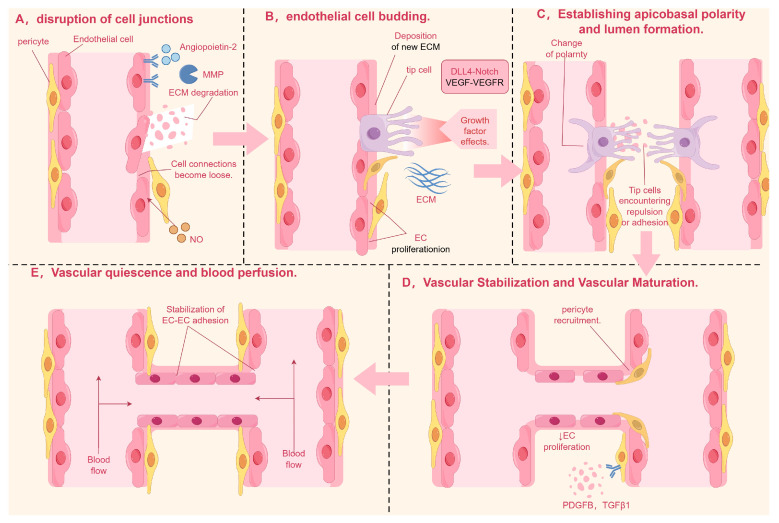
Note: (**A**) In the initial stage of angiogenesis, ECs respond to pro-angiogenic signals such as ANG-2 by activating MMPs, which disrupt intercellular tight junctions and confer migratory and motile capabilities to the cells. (**B**) ECs form bud-like structures under induction, differentiating into tip cells at the front and stalk cells at the rear. (**C**) EC polarization requires cytoskeletal reorganization, with molecules such as PAR3 and PKC promoting the formation of cell polarity and driving lumen development. (**D**) Vascular stabilization and maturation are mediated by PDGFB and TGFβ1, which recruit pericytes and vascular smooth muscle cells to adhere to the surface of newly formed vessels, providing support and stabilization. (**E**) In the final stage of angiogenesis, ANG-1 activates the TIE-2 receptor, stabilizing the vasculature and initiating blood flow. MMP, matrix metalloproteinase; ANG-1, angiopoietin-1; EC, endothelial cell; PDGFB, platelet-derived growth factor; TGFβ1, transforming growth factor-beta 1. By Figdraw.

Angiogenesis is a complex and dynamic cascade triggered by the disruption of junctions between endothelial cells (ECs). This intricate process involves the sprouting of ECs, the establishment of apical–basal polarity, the formation of vascular lumens, and the strengthening of vessel walls and ultimately leads to blood perfusion [1] (As shown in Figure 1). Impaired angiogenesis can lead to circulatory deficits and tissue necrosis, potentially triggering ischemic diseases. Conversely, excessive angiogenesis can result in pathological tissue proliferation, causing harm to healthy tissues and possibly leading to conditions such as atherosclerosis and rheumatoid arthritis.

In this article, we explore the molecular mechanisms underlying angiogenesis, investigate the impact of glycometabolism on this process, and synthesize the latest research advancements in therapeutic angiogenesis that utilize glycometabolic regulation. Our goal is to provide novel insights that may inform future research directions and the development of clinical applications in this field.

Glycolysis, a fundamental and central process in the body’s metabolic activities, plays a crucial role in angiogenesis. The initiation of angiogenesis hinges on the activation of glycolysis, while the maturation of new blood vessels is marked by a subsequent downregulation of this metabolic pathway [2]. Additionally, gluconeogenesis and other branches of glycolysis play significant roles in regulating the growth of endothelial cells, thereby influencing angiogenesis [3,4]. Atherosclerosis, retinal vascular diseases, and tumors, which are all angiogenesis-related diseases, are accompanied by endothelial cell (EC) glucose metabolism disorders. Gaining an in-depth understanding of the changes in EC glucose metabolism during angiogenesis is of great significance to the study of these diseases [5].

## 2. The Main Processes of Angiogenesis and Related Signaling Molecules

### 2.1. The Process of Angiogenesis

**Figure 2:**
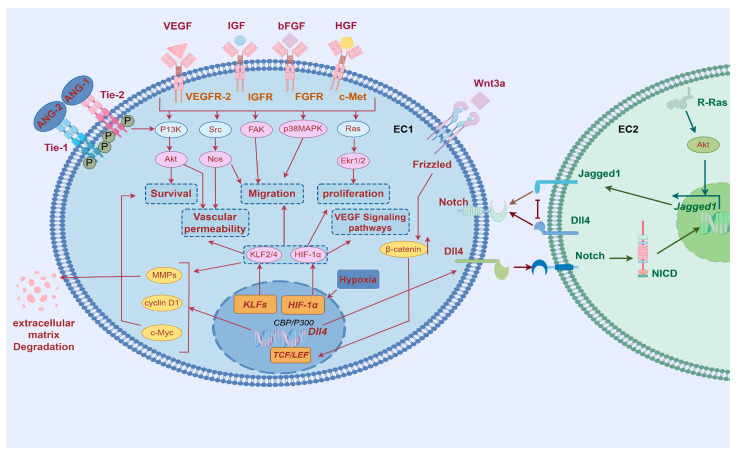
Regulatory mechanisms of angiogenesis. MMP, matrix metalloproteinase; ANG-1, angiopoietin-1; EC, endothelial cell; PDGFB, platelet-derived growth factor; TGFβ1, transforming growth factor-beta 10; VEGF, Vascular Endothelial Growth Factor; IGF, Insulin-like Growth Factor; bFGF, Basic Fibroblast Growth Factor; VEGFR-2, Vascular Endothelial Growth Factor Receptor-2; IGFR, Insulin-like Growth Factor Receptor; FGFR, Fibroblast Growth Factor Receptor; PI3K, Phosphoinositide 3-kinase; Akt, Protein Kinase B; Nos, Nitric Oxide Synthase; Ekr1/2, Extracellular signal-regulated kinases 1/2; HIF-1α, Hypoxia-inducible factor 1, subunit alpha; Notch, Notch receptor; Dll4, Delta-like ligand 4; Jagged1, Jagged Canonical Notch Ligand 1; Src, Src proto-oncogene, non-receptor tyrosine kinase; FAK, Focal Adhesion Kinase; P38MAPK, p38 Mitogen-Activated Protein Kinase; Ras, Rat Sarcoma; ANG-1, angiopoietin-1; ANG-2, angiopoietin-2; Tie-1, Tyrosine Kinase with Ig-like and EGF-like domains-1; Tie-2, Tyrosine Kinase with Ig-like and EGF-like domains-2. By Figdraw.

Angiogenesis, a continuous and dynamic process primarily orchestrated by endothelial cells, is initiated through precisely coordinated molecular events (As shown in Table 1 and Figure 2). Upon receiving pro-angiogenic signals such as angiopoietin-2 (ANG-2) at the vascular lumen surface, endothelial cells undergo the activation of matrix metalloproteinases (MMPs). These proteolytic enzymes subsequently degrade surrounding basement membrane proteins, leading to the destabilization of intercellular junctions and conferring enhanced migratory capacity to the endothelial cells [6].

The spatial patterning of endothelial cells during angiogenesis manifests through guided morphogenesis into distinct functional subtypes. Migratory tip cells, characterized by dynamic filopodial extensions, emerge at vascular sprouting fronts, while adjacent stalk cells provide structural stabilization through proliferative expansion. This cellular specialization is principally regulated by VEGF-VEGFR2 signaling axis activation [7] where ligand–receptor binding specificity triggers downstream signaling cascades via Notch-DLL4 lateral inhibition mechanisms and other evolutionarily conserved pathways governing cytoskeletal reorganization and mitotic programming [8].

Lumenogenesis requires cytoskeletal reorganization where apical junctions (VE-cadherin/β-catenin) redistribute circumferentially [9], while Filamentous Actin (F-actin) polymerization reinforces apical membranes [10]. VEGF activates Rho/actomyosin contractility via myosin light chain phosphorylation, driving apical constriction that transforms endothelial cells into polarized structures with functional luminal spaces through membrane domain specialization [11].

Subsequently, vascular stabilization and maturation are achieved through coordinated biological processes. Vascular stabilization involves the fortification of nascent vessel walls and reinforcement of inter-endothelial junctions, primarily mediated by platelet-derived growth factor B (PDGFB) and transforming growth factor-beta 1 (TGF-β1). These critical signaling molecules orchestrate the recruitment and subsequent adhesion of pericytes and vascular smooth muscle cells (VSMCs) to develop vasculature, thereby providing structural reinforcement and stabilizing nascent blood vessel architecture [12].

Once the newly formed vessel walls have achieved initial structural support through the stabilization phase, angiogenesis advances into a more complex maturation stage. This stage involves the coordinated actions of multiple cellular and molecular mechanisms to ensure the long-term stability and functional integrity of the vessels [13,14]. 

Vascular maturation encompasses two principal mechanisms: enhanced intercellular adhesion through the upregulated expression of cell adhesion molecules, facilitating vascular alignment and stabilization [15,16]; and structural reinforcement via pericyte-mediated extracellular matrix remodeling. Crucially, bidirectional crosstalk between pericytes and endothelial cells establishes a surveillance mechanism that monitors endothelial maturation status while promoting phenotypic transition to quiescence through paracrine signaling pathways [17]. This intricate cellular interplay ultimately facilitates the reconstruction of vascular homeostasis and the establishment of functional hemodynamic stability [18].

Finally, vascular quiescence and blood perfusion, which involve maintaining the quiescent phenotype of endothelial cells and initiating blood flow. After vascular maturation, mural cells release angiopoietin-1 (ANG-1) [19,20,21], which activates the TIE-2 receptor in endothelial cells (TEK tyrosine kinase recombinant protein receptor), promoting its phosphorylation and affecting the PI3K (phosphoinositide-3-Kinase) signaling pathway [22], thus promoting endothelial cell differentiation. ANG-1 and TIE-2 enhance endothelial cell connections support the actin cytoskeleton, maintain vascular stability, and promote endothelial cell survival and vascular barrier formation, as well as induce tissue perfusion, among other things [23].

In addition, pericytes can recruit myeloid bridge cells, which help newly formed blood vessels fuse with other vascular branches and initiate blood flow [24].

**Table 1:** Key regulatory factors and signaling pathways in angiogenesis.

Key Regulators and Signaling Pathways	Cellular Origins	Primary Functions	Antagonistic Proteins/Mechanisms	References
VEGF	Macrophages, endothelial cells, tumor cells, etc.	(1) Promote endothelial cell proliferation and filopodia extension (2) Promote extracellular matrix degradation and chemotaxis	The sFlt-1 (soluble VEGF receptor), bevacizumab (anti-VEGF mAb), VEGF-Trap (recombinant fusion protein)	[25]
bFGF	Fibroblasts, endothelial cells, etc.	(1) Promote the growth and differentiation of new cells on the wound surface (2) Induce the subcutaneous microvessel formation and improve the wound microcirculation	Heparanase, suramin (naphthaline polysulfonate), anti-FGF antibody	[26]
HGF	Interstitial cells, hepatic stellate cells, etc.	(1) Promote endothelial cell migration and proliferation (2) Stimulate the secretion of vasculocepoetin (3) Activation of angiogenic signals through the MAPK/PI3K pathway	NK4 (HGF antagonistic protein), SU11274 (c-Met inhibitor), anti-HGF antibody (Ficlatuzumab)	[27,28]
HIF-1	Various cells under hypoxic conditions	(1) Regulation of the cell cycle and DNA replication (2) Proproangiogenic chemokines and receptors (3) Transcriptional activation of VEGF and other angiogenic genes	PHD 2 (through oxygen-dependent degradation), HIF-1α inhibitors (PX-478, etc.)	[29,30]
KLFs	Endothelial cells, smooth muscle cells, etc.	(1) Regulation of endothelial cell migration and proliferation (2) Regulation of the endothelial cell metabolic pathway to promote angiogenesis	MiR-92a (by targeting KLF 2/4), KLF inhibitor (CRISPR knockout technique)	[30,31,32]
ANG-TIE signaling pathway	Endothelial cells, pericytes, etc.	(1) Promote the proliferation and differentiation of endothelial cells (2) Enhance the cell junctions and the actin cytoskeleton to maintain vascular stability	Angiopoietin inhibitor (AMG-386), TIE2 neutralizing antibodies	[22]
NOTCH-Dll 4 signaling pathway	Endothelial cells, hematopoietic stem cells, etc.	(1) Guide endothelial cell differentiation (2) Increase in VEGF receptor expression in the immature vascular plexus	γ-Secretase inhibitor (DAPT), anti-DLL4 antibody (Demcizumab)	[33,34,35]
Wnt/β-catenin signaling pathway	Endothelial cells, stem cells, etc.	(1) Regulation of endothelial cell proliferation and migration (2) Regulated vascular morphogenesis in coordination with the Notch pathway (3) Induced VEGF and other factors to indirectly affect angiogenesis	DKK 1 (Wnt inhibitor), ICG-001 (β-catenin inhibitor), AXIN (scaffold protein regulates β-catenin degradation)	[36,37]
Angiostatin	Proteolytic fragment of plasminogen	(1) Inhibition of the proliferation and migration of vascular endothelial cells (2) Block the pro-angiogenic effects of VEGF and FGF	Binding to endothelial cell surface ATP synthase, interfering with energy metabolism	[38,39]
Endostatin	The C-terminal fragment of collagen XVIII	(1) Inhibition of endothelial cell proliferation, migration, and survival (2) Induce endothelial cell apoptosis	Combining endothelial cell surface integrins (α5β1) and VEGFR2 to block the signaling pathway. Inhibition of the Wnt/β-catenin pathway	[40]
Platelet-reactive protein-1	Platelets, endothelial cells, and fibroblasts	(1) Direct inhibition of endothelial cell migration and angiogenesis (2) Activate the potential form of TGF-β, which indirectly regulates angiogenesis	Binding to the CD36 receptor to induce apoptotic signals (e.g., caspase-3 activation); antagonizing VEGF and FGF	[41]
Platelet factor 4	Platelet α particles	(1) Antagonize the pro-angiogenic effects of VEGF and FGF (2) Inhibition of endothelial cell proliferation and migration	Binding heparin-like molecules, blocking growth factor binding to the receptor	[41,42]

### 2.2. Angiogenesis and Vasculolysis Balance

Under normal physiological conditions, angiogenesis and vasculolysis are in a state of dynamic equilibrium. This balance ensures that tissues can generate blood vessels to meet the demands for oxygen and nutrients during wound healing and embryonic development while preventing excessive vessel formation. Therefore, the balance between angiogenesis and vasculolysis is crucial for the normal function of tissues and organs, and it is finely regulated by a variety of molecular mechanisms and signaling pathways [43,44]. Pro-angiogenic factors, such as VEGF, promote the proliferation, migration, and lumen formation of endothelial cells by binding to their receptors and activating downstream signaling pathways, thus supporting the formation of new blood vessels. In contrast, anti-angiogenic factors, such as thrombospondin-1 (TSP-1), inhibit the activity of endothelial cells by binding to surface receptors on endothelial cells or activating TGF-β1 complexes, preventing excessive blood vessel formation. Moreover, α-granules in activated platelets can release platelet factor 4 (PF4), which inhibits the proliferation and migration of endothelial cells, maintaining the homeostasis of the vascular system [45,46].

However, this balance is disrupted under pathological conditions. The consequences of excessive angiogenesis vary depending on the specific pathological environment, but it usually leads to tissue dysfunction, exacerbated inflammation, and disease progression. For example, tumor cells induce VEGF secretion to promote angiogenesis in order to meet their rapid growth requirements for oxygen and nutrients [47]. In diabetic retinopathy, excessive blood vessel formation can lead to abnormal vascular proliferation within the retina. These newly formed blood vessels are fragile and may result in retinal detachment and vision loss. On the other hand, when vasculolysis predominates, such as in chronic ischemic diseases, anti-angiogenic factors excessively inhibit the formation of collateral circulation [46]. In ischemic heart disease and peripheral vascular diseases, insufficient angiogenesis leads to tissue hypoxia and necrosis. Regulating the balance between angiogenesis and vasculolysis can promote the formation of collateral circulation and improve tissue perfusion. Therefore, the combined application of anti-VEGF drugs and pro-angiogenic therapies (such as HGF) has the potential for clinical application as it can both inhibit pathological angiogenesis and promote functional vascular repair [48].

## 3. Glucose Metabolism in Angiogenesis

ECs exhibit a glycolytic-dominant metabolic phenotype, with glycolysis satisfying >85% of their ATP demand [49]. Compared to other cell types, ECs have relatively fewer mitochondria, and the proportion of ATP generated through aerobic oxidation is smaller. Thus, despite direct contact with oxygen in the bloodstream, quiescent ECs consume less oxygen, while other cells preferentially utilize oxygen for aerobic oxidation to produce energy [50,51]. Additionally, by relying on glycolysis, ECs reduce their oxygen consumption, thereby allowing more oxygen to be transferred to surrounding cells [52].

### 3.1. Glycolysis Is a Primary Metabolic Pathway That Regulates Angiogenesis

Current research indicates that glycolysis plays a pivotal role in angiogenesis. During sprouting, tip cells migrate into hypoxic microenvironments where they cannot rely on oxidative metabolism and must instead utilize glycolysis [53]. Glycolysis rapidly generates ATP, enabling ECs to swiftly form highly motile and fast-moving lamellipodia and filopodia under hypoxic conditions [49]. Glycolysis-dominated EC metabolism not only supplies energy but also interacts with various angiogenic signaling mechanisms to comprehensively regulate cellular behaviors across all stages of angiogenesis [52].

Moreover, key glycolytic enzymes such as PFKFB3, PKM2, and HK2 support the mi-gration of tip cells and the proliferation of stalk cells by regulating glycolysis, thereby driving vascular sprouting [53], and LDHA promotes cell proliferation and migration in VSMCs by regulating lactate production, contributing to neointimal formation [51]. Metabolic products like lactate and pyruvate promote angiogenesis by regulating the extracellular acidic environment and activating relevant signaling pathways, such as the HIF-1α pathway [54,55]. Signaling pathways such as VEGF and AMPK promote EC migration and angiogenesis by regulating glycolysis and mitochondrial respiration [30].

### 3.2. GLUTs

Glucose transporters (GLUTs) are crucial for the transport of glucose into cells. Among them, GLUT1/3 are mainly responsible for glucose uptake into endothelial cells and are essential for endothelial cell glycolysis. Factors upstream of glycolysis, such as HIF-1α and mTORC1, can enhance glycolysis by increasing the expression of GLUT1/3 [56,57]. Glycolysis is the primary bioenergetic pathway in ECs, accounting for more than 85% of the ATP supply within the cell. Simulating angiogenesis conditions in vitro through VEGF stimulation can increase GLUT1 expression and enhance glycolysis. The absence of EC-GLUT1 reduces endothelial energy availability and proliferation but does not affect migration, thereby delaying developmental angiogenesis [58]. In a hindlimb ischemia model, ischemic training can upregulate HIF-1α and GLUT-1, improving blood flow recovery and arteriogenesis after permanent hindlimb ischemia. At present, GLUTs in endothelial cells have been confirmed to be involved in processes such as angiogenesis, but their specific mechanisms of action have not been fully elucidated [59]. Studying how endothelial glucose transporters regulate angiogenesis is necessary for finding a new therapeutic target for the treatment of ischemic diseases [60,61].

### 3.3. HK2

Hexokinase 2 (HK2) is a rate-limiting enzyme in glycolysis, phosphorylating glucose to produce glucose-6-phosphate. bFGF can promote the expression of the proto-oncogene MYC, thereby regulating HK2 levels and glycolysis. In the absence of FGF signaling, decreased HK2 levels lead to reduced glycolysis, impairing endothelial cell proliferation and migration and affecting vascular maturation [62]. The transcription factor Foxp1 plays a regulatory role in the HIF-1α-HK2 pathway, which can improve the high glycolysis of tumor ECs and limit tumor angiogenesis, thereby effectively slowing down tumor growth [63]. Reducing glycolysis through HK2 inhibition can suppress spiral artery remodeling, increase mitochondrial membrane permeability, and induce endothelial cell apoptosis. These findings underscore the critical role of HK2 in glycolytic metabolism and its impact on endothelial cell function [64,65].

### 3.4. PFKFB3

PFKFB3 catalyzes the synthesis of fructose-2,6-bisphosphate (F-2,6-BP), thereby upregulating the activity of fructose-2,6-bisphosphatase and acting as a key rate-limiting enzyme in endothelial glycolysis. This enzyme is regulated by a variety of signaling molecules that influence angiogenesis. For example, laminar shear stress impacts angiogenesis through PFKFB3’s response to KLF2 signaling, which in turn upregulates glycolysis in endothelial cells. This upregulation activates signaling pathways such as VEGF, promoting endothelial cell proliferation and angiogenesis [66].

During the sprouting phase, HIF-1α is translationally activated in response to hypoxia, which then downregulates the expression of VE-cadherin in endothelial cells via PFKFB3, affecting endothelial cell motility and angiogenesis [67,68,69]. During the migratory phase of EC motility, F-actin interacts with PFKFB3 to augment its enzymatic activity, thereby elevating glycolytic flux and ATP production. This interaction is pivotal for the assembly of filopodia and lamellipodia, which are critical protrusive structures for EC migration and angiogenesis. The suppression of PFKFB3 expression compromises the formation of these cellular extensions, thereby hindering EC movement and the progression of angiogenesis [70,71].

In the process of promoting the stem cell phenotype by Notch signal transduction, the expression of PFKFB3 is inhibited, reducing glycolysis-related factors and thereby decreasing the competitiveness of tip cells. The knockdown of PFKFB3 inhibits the activity of both terminal cells and stalk cells, affecting vascular sprouting [72,73]. Angiogenic factors such as FGF and VEGF can also upregulate the expression of PFKFB3, promoting the generation and selection of vascular tip cells [4,73]. However, the product F-2,6-BP catalyzed by PFKFB3 can initiate the VEGF-DLL4-Notch signaling pathway, promoting tip cell competition and affecting the embryonic process of endothelial cells. The feedback pathways involved in this process are not well studied. The current understanding reveals that PFKFB3 and selective angiogenic signaling molecules are engaged in a feedback regulatory mechanism. Specifically, once activated by PFKFB3-driven glycolysis, VEGF and eNOS can reciprocally upregulate PFKFB3 expression, establishing a positive feedback loop that amplifies the angiogenic response [74]. This positive feedback mechanism ensures the continuity and effectiveness of the angiogenesis process while also highlighting the complexity and significance of PFKFB3 in the regulation of angiogenesis.

### 3.5. PKM2

Pyruvate kinase (PK) is instrumental in the glycolytic pathway, catalyzing the conversion of phosphoenolpyruvate (PEP) into pyruvate and ATP. Pyruvate kinase M2 (PKM2), a predominant isoform of PK, participates not only in cytoplasmic glycolysis but also translocates to the nucleus in a dimeric configuration, where it functions as a coactivator for transcription factors and modulates gene expression. Within the nucleus, PKM2 interacts with a spectrum of transcription factors, including Jmjd4, RAC1, β-catenin, and HIF-1α, thereby influencing gene expression and promoting angiogenesis. The activation of PKM2 also regulates glycolysis and mitochondrial dynamics such as fission and fusion and subsequently enhances the vasculogenesis of very small embryonic-like progenitor cells (VSEL-PCs) [75]. PKM2 secreted by neutrophils has also been found to play a role in tissue regeneration and wound healing by promoting the germination of new blood vessels [76]. In hypoxic pancreatic tumors, PKM2 interferes with NF-κB/p65 and HIF-1α activation, ultimately triggering VEGF-A expression to promote vascularization [77]. PKM2 is also involved in angiogenesis both in vitro and in vivo by providing ATP to regulate VE-cadherin internalization and EC linkage [78].

### 3.6. LDHA

Lactate dehydrogenase A (LDHA) catalyzes the conversion of pyruvate to lactate in glycolysis. Physiologically, rapidly proliferating cells rely on glycolysis for growth, resulting in lactate accumulation and angiogenesis [79]. Under normoxic conditions, pulmonary microvascular endothelial cells demonstrate high glucose uptake and significant lactate production, leading to the upregulation of GLUT-1, HK2, and LDHA, which are essential for sustaining endothelial cell proliferation. In pathological contexts, lactate is taken up by tumor-associated macrophages, triggering the activation of G protein-coupled receptors and adenylate cyclase. This activation induces signaling molecules such as CD206 and VEGF, initiating the sprouting of new blood vessels from tip cells. Tumor-derived lactate also stimulates the activation of factors like VEGF, Ang-1, and IL-8 in ECs through the PI3K/Akt signaling pathway. This activation regulates the expression of cyclin-D1, a key player in the cell cycle, prompting DNA replication and EC mitosis. Consequently, EC sprouting is activated, leading to the formation of new blood vessels [80]. Additionally, lactate can promote angiogenesis by activating HIF-1α and inducing the expression of various pro-angiogenic factors [81,82]. As previously highlighted, HIF-1α serves as an upstream regulator of glycolysis, with lactate further activating HIF-1α to stimulate glycolysis, potentially leading to increased lactate production. The sustained accumulation of lactate can also drive histone lactylation, which is crucial in VEGF-induced angiogenesis. In this context, a feedback loop involving H3K9la and HDAC2 is essential. Consequently, lactate, produced in high quantities through glycolysis, is not merely a metabolic byproduct but also a promoter of continued glycolysis and a pivotal factor in the advancement of angiogenesis [83]. This underscores the dual function of lactate in metabolic regulation and the facilitation of angiogenic processes. However, the complex and dynamic interrelationships involved are not yet fully understood or systematically explained.

### 3.7. Other Roles of Glucose Metabolism in Angiogenesis

ECs also utilize other glucose metabolic pathways; however, their exact roles in angiogenesis have not been fully proven, especially in vivo.

Initially, when glucose is phosphorylated by HK2 to form glucose-6-phosphate, glucose-6-phosphate can enter the oxidative branch of the pentose phosphate pathway (oxPPP) to generate ribulose-5-phosphate and NADPH [84]. The inhibition of either the oxPPP or non-oxidative (non-oxPPP) branches of the pentose phosphate pathway, which are governed by the rate-limiting enzymes glucose-6-phosphate dehydrogenase and transketolase, respectively, results in compromised EC activity and migration. The oxidative arm of the PPP is essential for the establishment of vascular mural cell (vMC) coverage in ECs and plays a role in promoting vascular maturation by regulating the deposition of the vascular basement membrane and the interactions between ECs and vMCs [85].

Secondly, the hexosamine biosynthetic pathway (HBP), a branch of glycolysis, originates from fructose-6-phosphate (F6P) and is responsible for the synthesis of uridine diphosphate N-acetylglucosamine (UDP-GlcNAc). Under nutritional stress conditions, glucose levels act as triggers for the activation of the HBP. This pathway helps maintain the homeostatic balance of glucose metabolism by modulating other glucose metabolic pathways through the key regulatory molecule O-linked N-acetylglucosamine (O-GlcNAc) [86]. Fructose-6-phosphoamidinoamidinyltransferase 1 (GFAT1) is a rate-limiting enzyme of the HBP and a negative regulator of angiogenesis [87]. VEGF inhibits angiogenesis through the AMP-activated protein kinase (AMPK)-mediated phosphorylation of GFAT1 in ECs [88]. Therefore, the HBP is considered a nutrient sensor that regulates angiogenesis, and its glycosylation state determines the function of the angiogenesis pathway [89].

Gluconeogenesis is the metabolic pathway responsible for the synthesis of glucose from non-carbohydrate sources, involving reactions that are reversible in the glycolytic sequence. Phosphofructokinase (PFK), the most critical regulatory enzyme in this process, intensifies the negative regulation of glycolysis during gluconeogenesis, thereby influencing endothelial cell function and suppressing angiogenesis. Phosphoenolpyruvate carboxykinase 1 (PCK1), a rate-limiting enzyme in gluconeogenesis, plays an essential role in angiogenesis, and its absence can detrimentally affect endothelial cell function. PCK1 is posited to stimulate angiogenesis by modulating the expression of Gαi3 and the activation of the Akt pathway [90]. Fructose 1,6-bisphosphatase 1 (FBP1), another rate-limiting enzyme of gluconeogenesis, acts as a negative regulator of glycolysis and affects angiogenesis. Retinoic acid inhibits angiogenesis by promoting FBP1-driven gluconeogenesis and reducing glycolytic flux [3].

## 4. Therapeutic Strategies Targeting Angiogenesis

### 4.1. Therapeutic Angiogenesis

Ischemic diseases, particularly peripheral arterial disease (PAD) and myocardial infarction (MI), are significant causes of high morbidity and mortality. Common methods for treating ischemic diseases in clinical practice include drug therapy, angioplasty and stent implantation, bypass graft surgery, and thrombolytic therapy [91,92,93]. However, these traditional treatments have limitations in some cases and are not suitable for all patients (As shown in Table 2).

Therapeutic angiogenesis is a treatment strategy that promotes neovascularization to improve blood flow in ischemic tissues. In ischemic diseases, insufficient blood supply leads to tissue hypoxia and damage. Therefore, promoting angiogenesis to restore blood flow is an important means of treating these diseases [94]. For example, angiogenesis contributes to cardiac function recovery after acute myocardial infarction (AMI) [95,96]. The advantage of therapeutic angiogenesis lies in its ability to specifically promote angiogenesis in ischemic areas, thereby improving local blood flow. Additionally, it is usually less invasive compared to traditional surgery, being more gentle and safer. Moreover, this treatment method has the potential for long-term efficacy, as it can stimulate the body’s own angiogenic capacity, achieving sustained blood flow improvement. Furthermore, it not only promotes blood flow but also facilitates the repair and regeneration of damaged tissues. Finally, therapeutic angiogenesis can be combined with traditional treatment methods to provide more personalized and comprehensive treatment plans, offering patients more treatment options and better outcomes [97].

**Table 2:** Advantages and disadvantages of common clinical treatments for ischemic diseases.

Therapeutic Method	Mechanism of Action	Superiority	Inferior Strength or Position	Clinical Stage/Representative Studies	Reference
Angioplasty, bypass surgery	Mechanical expansion of the narrow vessel or establishment of bypass channels to restore blood flow	(1) Significant efficacy (postoperative blood flow recovery rate > 80%) (2) Fast recovery (3 days)	(1) Only for large vessel lesions (2) Postoperative restenosis rate (20–30%) (3) It is not suitable for diffuse microvascular lesions	Clinical routine application Ultrasound guidance optimization (improve precision and reduce complications)	[98]
Proangiogenic factor therapy	Neovascularization was stimulated by factors such as VE GF and bF GF	(1) Minimally invasive (local injection/gene delivery) (2) Inoperable microvascular lesions (such as Buerger’s disease)	(1) High cost (single session > USD 5000) (2) Susceptibility (continuous administration requirements) (3) Risk of excessive vascular proliferation (edema, tumor)	Clinical Phase II trial Synthetic mRNA-VE GF (improved collateral circulation in 74% of patients)	[93,99,100]
Medication	Multi-target regulation (anti-inflammatory, pro-angiogenic, improved metabolism)	(1) Systemic action (covering the extensive ischemic area) (2) Easy use for oral/intravenous administration	(1) Poor targeting (systemic side effects) (2) Low delivery efficiency (<10% to the target tissue) (3) Long-term medication is required (low compliance)	Preclinical/early-stage clinical setting Nanoparticle delivery system (improves targeting) Exosomal drug loading	[97,101]
Stem-cell/exosome therapy	Promote angiogenesis and tissue repair through paracrine factors (e. g., exosomal miRNA)	(1) Noninvasive cell repair (2) Regulation of the immune microenvironment (3) Long-acting effect (a single injection effect lasts for 4 weeks)	(1) Difficulties in standardization (high heterogeneity) (2) Potential tumorigenicity (3) High storage and transportation costs	Clinical Phase I/II trial Umbilical cord mesenchymal stem cell exosomes (60% increase in wound healing rate)	[102,103,104,105,106]
Ultrasound targeted therapy	Low-frequency ultrasound combined with microbubbles enhances drug penetration or directly induced vasodilation	(1) Non-invasive (2) Collaborative drug therapy (3–5 times higher efficiency)	(1) High equipment dependence (2) Long-term safety is waiting to be verified (3) Limited effect on deep organization	Clinical exploration stage VE GF gene (40% increase in blood flow recovery in animal model)	[72]
Platelet-rich blood plasma (PRP) therapy	Improving the immune environment of ischemic tissue by stimulating endothelial cell proliferation and migration with high concentrations of growth factors	Reduces immunogenicity (avoid the problem of immune response in traditional gene therapy) Can cooperate with pro-angiogenic factors	The cost is higher The PRP preparation process is complicated Long-term safety is to be verified	Preclinical/early-stage clinical setting Cord blood-derived PRP gel (reducing wound area by 80% within 14 days)	[26,107,108]

### 4.2. New Strategies for Targeted Metabolism

In recent years, VEGF therapy has shown drawbacks, such as side effects, limited efficacy, drug resistance, and other intractable complex challenges. Interventions targeting metabolism, which regulates the metabolism of ECs and other cells, have emerged as a promising alternative to traditional VEGF treatment, especially in the treatment of cardiovascular diseases, tumors, and ischemic diseases.

Inhibitors of PFKFB3 reduce glycolytic activity and lactate production, thereby suppressing angiogenesis. They are applied to block some glycolysis processes and inhibit pathological angiogenesis. For example, the PFKFB3 inhibitor 3PO effectively inhibits choroidal neovascularization in mouse ECs by reducing glycolytic activity [73]. The effects of 3PO on tumors are similar; however, it is important to note that 3PO does not bind to PFKFB3 [109]. Its specificity is relatively poor, and the high dosage requirements lead to its delay in entering the clinical trial phase. Consequently, an inhibitor that exhibits strong affinity for PFKFB3, PFK15, has emerged [110]. The synergistic inhibitory effect of PFK15 and the multi-kinase inhibitor sunitinib on the proliferation and migration of human umbilical vein endothelial cells (HUVECs) has been studied. The research results indicate that combining the inhibition of glycolytic activity in endothelial cells with the blockade of growth factor receptors may be a promising anti-angiogenic therapeutic approach [111]. In the treatment of infantile hemangiomas, the combination therapy of PFK15 and propranolol has shown significant effects [110]. The combination therapy of PFK15 is gaining attention in the medical field. Moreover, the benzoxindole derivative, along with the 6-phosphofructo-2-kinase/fructose-2,6-bisphosphatase 3 inhibitor (AZ-67), has accumulated substantial evidence in basic research [112]. Anti-glycolytic PA compounds have been shown to potentially affect several steps involved in angiogenesis [113]. PFK-158 is currently undergoing Phase I clinical trials. Compared to 3PO and PFK15, PFK-158 has shown higher selectivity and efficacy and has demonstrated better tolerability in animal models. Moreover, several studies have confirmed the significant effects of PFK-158 in anti-tumor and anti-angiogenesis activities [114].

While glycolytic inhibitors have advanced into clinical trials for cancer therapy, their application in cardiovascular diseases remains in nascent stages. Preclinical studies suggest that glycolytic modulation may offer novel therapeutic avenues for myocardial injury, though the inhibition of glucose oxidation yields context-dependent outcomes. For instance, the localized delivery of 2-deoxyglucose (2-DG), a hexokinase inhibitor, via composite patches in murine left anterior descending artery ligation models significantly attenuated post-infarction glycolysis and demonstrated cardioprotective effects [115]. Secondly, vascular endothelial cells, macrophages, and fibroblasts rely on glycolysis to produce ATP and biosynthesis. The complete and permanent inhibition of glycolytic enzymes may have harmful consequences for heart function. Enhancing OXPHOS in macrophages to promote anti-inflammatory/repair functions, while inhibiting OXPHOS in fibroblasts, suggests that metabolic intervention has cell-type specificity [116]. Glycolysis is enhanced during ischemia, so targeting glycolysis and glucose utilization in cardiomyocytes may have positive effects [117]. However, experiments have also confirmed that Pkm2 deletion can induce cardiomyocyte proliferation and improve cardiac structure and function after MI, indicating that different metabolic strategies should be chosen according to different pathological stages [118].

Targeting fatty acid oxidation (FAO) not only disrupts bioenergetic substrate flux but also transcriptionally represses angiogenic signaling cascades, positioning it as a dual-mechanism strategy to combat VEGF-driven vascular pathologies [5,119,120].

Amino acid metabolism, particularly the serine synthesis pathway, plays a significant role in angiogenesis. Key enzymes in this pathway, such as phosphoglycerate dehydrogenase (PHGDH), are essential for maintaining the redox balance in endothelial cells and promoting angiogenesis [121,122]. Inhibiting the synthesis of glutamine or the activity of glutaminase 1 (GLS1) can disrupt the cellular redox status, thereby impairing angiogenesis [123].

## 5. Discussion

In the treatment of ischemic diseases, targeting glycolysis regulation to promote angiogenesis has shown significant potential. Hypoxia, a core pathological feature of ischemic injury, activates the expression of glycolysis-related angiogenesis genes through signaling axes such as HIF-1α, providing rapid energy support for angiogenesis. It also mediates epigenetic remodeling through metabolic products such as lactate and α-ketoglutarate (e.g., histone lactylation), synergistically regulating key pathways such as VEGF and Notch and promoting endothelial cell proliferation, migration, and vascular network formation. Studies have shown that specifically enhancing endothelial cell glycolysis flux (e.g., by activating the PFKFB3 kinase) can significantly improve the angiogenic capacity of ischemic tissues, while inhibiting key glycolytic enzymes leads to angiogenic defects, highlighting the regulatory role of glycolysis in angiogenesis.

This article provides a comprehensive review of angiogenesis and its metabolic regulatory mechanisms, with a focus on recent advancements in therapeutic angiogenesis that exploit metabolic regulation. Metabolism exerts a multifaceted and intricate influence on angiogenesis, involving the regulation of key enzyme activities, alterations in signal transduction pathways, and the dynamic interplay between various metabolic pathways. Despite these insights, research in this field remains in its early stages. Future investigations should prioritize the identification and validation of key metabolic targets to further elucidate the complex interplay between metabolic pathways and angiogenic signaling networks. Such efforts may unveil novel therapeutic strategies for ischemic diseases.

In recent years, therapeutic strategies targeting glycolysis intervention have entered the clinical translation stage. For instance, by using small-molecule drugs or gene editing techniques to target glycolysis metabolic nodes (such as the dynamic balance of PKM2 isoforms and PFKFB3 activity), the functional state of endothelial cells can be precisely regulated. On the other hand, metabolic precursors delivered by nanocarriers (such as pyruvate and glutamine) promote the establishment of collateral circulation by reshaping the metabolic microenvironment in ischemic areas. However, current research still faces multiple challenges: (1) The interaction mechanism between the glycolysis network and angiogenesis signaling pathways has not been fully elucidated, especially the non-classical functions of metabolic enzymes (such as the protein kinase activity of PKM2) in angiogenesis regulation, which still need an in-depth exploration. (2) How the dynamic balance between different glycolytic branches (glycolysis, the pentose phosphate pathway, and the hexosamine pathway) affects the angiogenic phenotype remains to be clarified. (3) The lack of spatiotemporal specificity in metabolic intervention may lead to excessive or ectopic angiogenesis, such as pathological angiogenesis or tumor vascularization risks.
